# Insights into the Differential Composition of Stem-Loop Structures of Nanoviruses and Their Impacts

**DOI:** 10.1128/spectrum.04798-22

**Published:** 2023-06-27

**Authors:** Aamir Lal, Amen Shamim, Eui-Joon Kil, Thuy T. B. Vo, Muhammad Amir Qureshi, Nattanong Bupi, Marjia Tabassum, Sukchan Lee

**Affiliations:** a Department of Integrative Biotechnology, Sungkyunkwan University, Suwon, South Korea; b Department of Plant Medicals, College of Life Sciences, Andong National University, Andong, South Korea; c Agricultural Science and Technology Research Institute, Andong National University, Andong, South Korea; d Department of Computer Science, University of Agriculture, Faisalabad, Pakistan; Fujian Agriculture and Forestry University

**Keywords:** multipartite viruses, nanoviruses, molecular dynamics simulation, common region-stem-loop

## Abstract

Multipartite viruses package their genomic segments independently and mainly infect plants; few of them target animals. *Nanoviridae* is a family of multipartite single-stranded DNA (ssDNA) plant viruses that individually encapsidate ssDNAs of ~1 kb and transmit them through aphids without replication in aphid vectors, thereby causing important diseases in host plants, mainly leguminous crops. All of these components constitute an open reading frame to perform a specific role in nanovirus infection. All segments contain conserved inverted repeat sequences, potentially forming a stem-loop structure and a conserved nonanucleotide, TAGTATTAC, within a common region. This study investigated the variations in the stem-loop structure of nanovirus segments and their impact using molecular dynamics (MD) simulations and wet lab approaches. Although the accuracy of MD simulations is limited by force field approximations and simulation time scale, explicit solvent MD simulations were successfully used to analyze the important aspects of the stem-loop structure. This study involves the mutants’ design, based on the variations in the stem-loop region and construction of infectious clones, followed by their inoculation and expression analysis, based on nanosecond dynamics of the stem-loop structure. The original stem-loop structures showed more conformational stability than mutant stem-loop structures. The mutant structures were expected to alter the neck region of the stem-loop by adding and switching nucleotides. Changes in conformational stability are suggested expression variations of the stem-loop structures found in host plants with nanovirus infection. However, our results can be a starting point for further structural and functional analysis of nanovirus infection.

**IMPORTANCE** Nanoviruses comprise multiple segments, each with a single open reading frame to perform a specific function and an intergenic region with a conserved stem-loop region. The genome expression of a nanovirus has been an intriguing area but is still poorly understood. We attempted to investigate the variations in the stem-loop structure of nanovirus segments and their impact on viral expression. Our results show that the stem-loop composition is essential in controlling the virus segments' expression level.

## INTRODUCTION

Single-stranded DNA (ssDNA) viruses are the smallest capsid-encoding pathogens known to infect eukaryotic organisms. Circoviruses (1, 2), bidensoviruses (3), small circular viruses (4), redondoviruses (5), anelloviruses (6, 7), genomoviruses (8), and circular replication-associated protein (Rep)-encoding single-stranded (CRESS) DNA viruses (9, 10) are some of the important ssDNA viruses that infect animals, silkworms, humans, fungi, insects, and marine invertebrates. Monopartite and bipartite viruses are prevalent among these ssDNA viruses, with one and two segments. Some viruses are multipartite and have two or more segmented genomes packaged into separate virions capable of propagating independently (11, 12). Based on their genomic organization, the International Committee on Taxonomy of Viruses categorized ssDNA plant viruses into two families: (i) *Geminiviridae* (13) and (ii) *Nanoviridae* (11).

**FIG 1:**
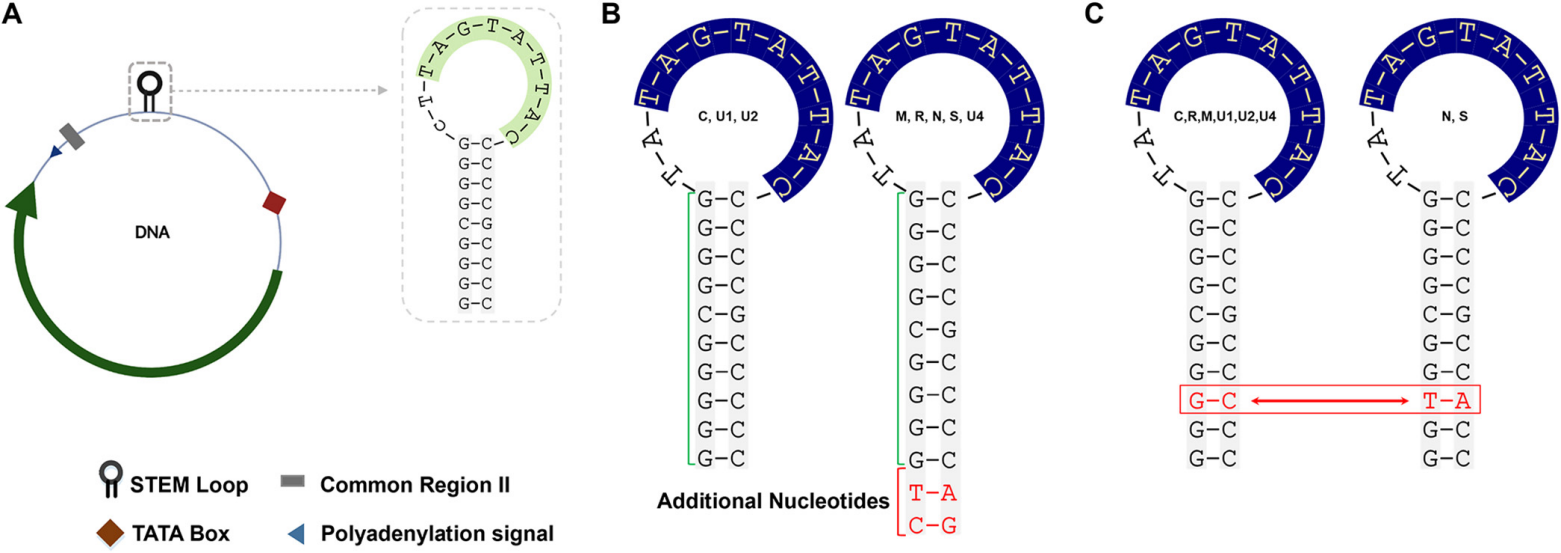
Structure of a nanovirus DNA segment and variations in the stem-loop region of MDV. (A) Structure of a nanovirus segment with an ORF and IR. The stem-loop in IR possesses nonanucleotide TAGTATTAC, where the nicking site lies and starts the replication. (B) Image depicting the variations in the neck region of the stem-loop based on the length of the base pairing (9/11 base pairing); (C) image showing the variations in the nature of base pairing (T-A/G-C) at specific position 7 in the neck region of the stem-loop region among the segments.

*Nanoviridae* has been categorized into two genera (*Nanovirus* and *Babuvirus*) based on their genome organization and transmission vectors, along with the categorization of coconut foliar decay virus as an unassigned species (14). Nanoviruses are nonenveloped with icosahedral and round geometries and T=1 symmetry with a diameter of 18 to 19 nm. Nanoviruses are multipartite viruses with 8 to 10 circular ssDNA components of ~1 kb (15, 16). Babuviruses contain six components of ~1 to 1.1 kb (17). All of these components are encapsidated separately into individual virions, each with a specific role (18, 19): i.e., DNA R encodes the master replication initiator protein (20, 21), DNA C encodes the cell cycle-link protein (22), DNA M encodes the movement protein, DNA S encodes the capsid protein (23), and DNA N encodes the nuclear shuttle protein (16, 23, 24). Despite numerous attempts to investigate DNAs U1, U2, and U4 of nanoviruses and U3 of babuviruses and the satellite molecules associated with nanoviruses, their biological functions remain obscure. All segments contain conserved inverted repeat sequences, potentially forming a stem-loop structure within a common region–stem-loop (CR-SL) and a conserved nonanucleotide, TAGTATTAC (Fig. 1A). Nanoviruses replicate through a rolling circle mechanism, and to initiate replication, the viral Rep nicks the conserved nonanucleotide sequence within the stem-loop structure (16, 25). This study investigated the variations in the stem-loop structure of the nanovirus segments and their impact on gene expression.

We leveraged experimental measurements of nanovirus segments to test the variation in structures by the molecular dynamics (MD) simulation to determine the structural stability of stem-loop segments that are in agreement with the experiments. Even though the accuracy of MD simulation is limited by force field approximations and simulation time scale, explicit solvent MD simulations were successfully used to analyze important aspects of the stem-loop structure. We analyzed insights into nanosecond dynamics of the stem-loop structures’ cation binding, hydration, and base pairing. Therefore, this study aimed to determine the variation of stem-loop segments, their stem-loops, and their effects on gene expression.

## RESULTS

### Sequence analysis and segment characterization.

**FIG 2:**
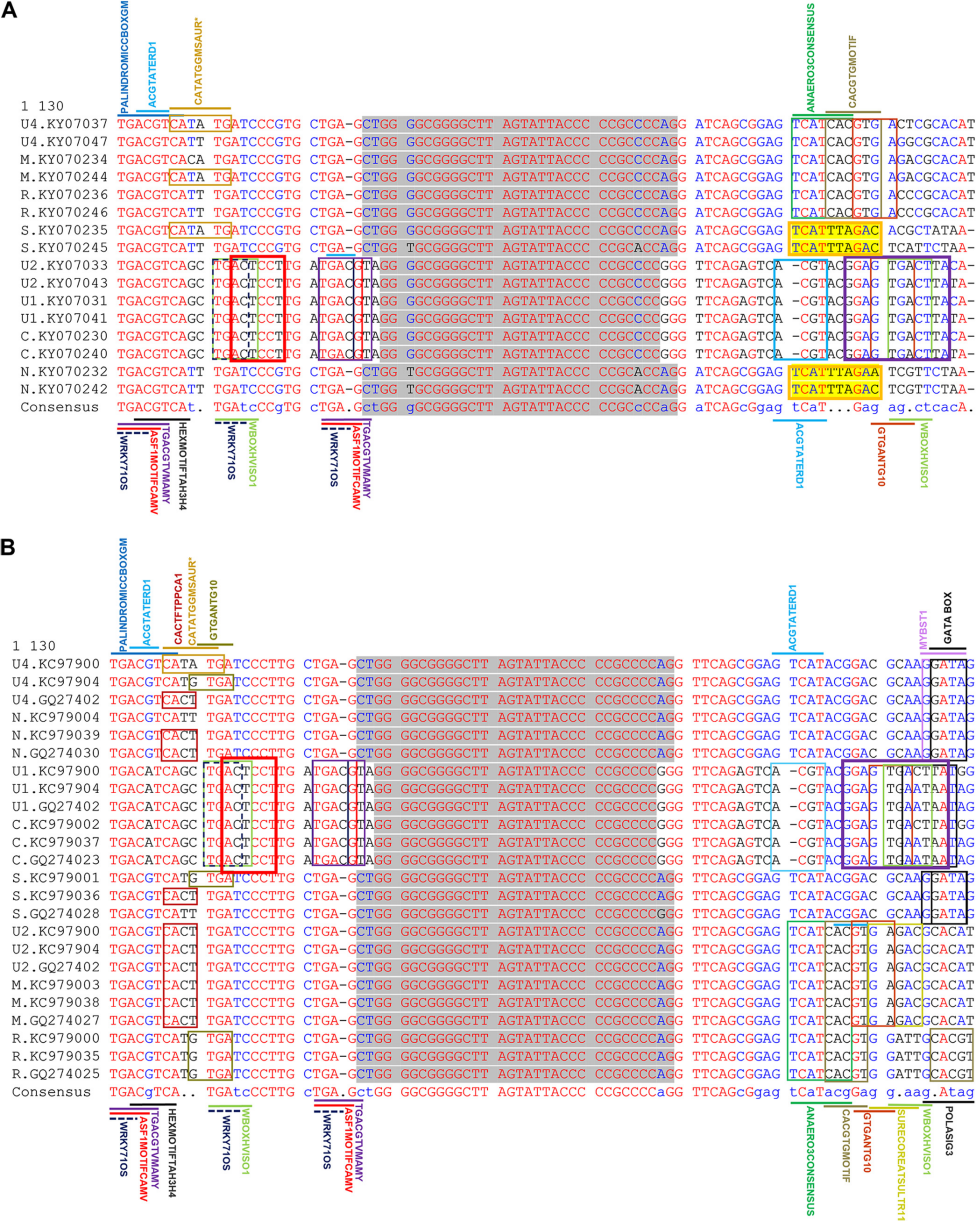
Analysis of IR based on motif composition in the SL-CR of all segments of MDV and FBNYV. (A) Motifs in the SL-CR of MDV using the PLACE software. Motifs in DNAs C, U1, and U2 are similar, as WBOXNTERF3, GTGANTG10, and ACGTATERD1 constitute only these three segments. N and S segments consist of exactly the same region as highlighted (with no data available yet regarding this region in yellow). (B) Among FBNYV segments, DNAs C and U1 possess the same composition of motifs in the SL-CR. WBOX, WRKY710S, ASF1MOTIFCAMV, TGACGTVMAMMY, and GTGANTG10 are found only in DNA C and U1 segments.

When aligned separately, all segments of each nanovirus showed that CR-SL in IR is the most conserved region among all segments. In the CR-SL of milk vetch dwarf virus (MDV) segments, DNAs C, U1, and U2 had different lengths of the stem-loop (neck region), with 9-nucleotide (nt) base pairings, than DNAs R, S, M, N, and U4, with 11-nt base pairings in the stem-loop (Fig. 1B). We also noticed at position 7 of the nucleotide base pairings, nucleotide pairing was different in DNAs N and S (T-A) than in DNAs R, C, M, U1, U2, and U4 (G-C) in the stem-loop structure (Fig. 1C). An intriguing aspect to notice is the expression level of DNAs C, U1, and U2 (all with 9-nt base pairings in the stem-loop) was almost same whereas, the expression level of DNAs N and S (which contains T-A at position 7) was also same when analyzed through quantitative PCR (qPCR) in the papaya sample infected with MDV. These variations in the length of the stem-loop neck region and nucleotide pairing were observed among segments in other nanoviruses as well: e.g., faba bean necrotic stunt virus (FBNSV), faba bean necrotic yellows virus (FBNYV), black medic leaf roll virus (BMLRV), and pea necrotic yellow dwarf virus (PNYDV) (Table 1; see Fig. S1A to G in the supplemental material). Due to these variations, motif formation among the segments varies, as shown for MDV and FBNYV (Fig. 2A and B).

**TABLE 1:** Type of variations in the stem-loop region of DNA segments of nanoviruses^*a*^

Species	DNA segment(s) with length difference of:		DNA segment(s) with nt substitution(s)		Site no.	nt change(s)
	11 nt	9 nt	Major	Mutant		
Milk vetch dwarf virus	M, R, N, S, U4	C, U1, U2	C, R, U1, U4, M, U2	N, S	7	G-C→T-A
Faba bean necrotic stunt virus	M, R, N, S, U2, U4	C, U1	M, R, N, S, U2, U4	C, U1	6	G-C→T-A
Faba bean necrotic yellows virus	M, R, S, N, U2, U4	C, U1	M, R, N, S, U2, U4, C, U1	X	X	X
Black medic leaf roll virus	X	M, R, N, S, C, U2, U4, U1	M, R, S, U2, U4, C, U1	N	6	G-C→A-T
Pea necrotic yellow dwarf virus	M, R, N, S, C, U1, U2	U4	M, R, N, S, C, U1, U2	U4	5	C-G→G-C
					7	C-G→G-C
Faba bean yellow leaf virus	M, R, S, C, N, U1, U2, U4	X	M, R, C, U1, U2, U4	S, N	5	G-C→A-T
					6	G-C→C-G
Subterranean clover stunt virus	X	M, R, S, C, N, U1, U2, U4	M, R, N, U1, U2, U4	S, C	6	A-T→G-C
					7	C-G→G-C
Cow vetch latent virus	M, R, N, S, C, U1, U2	U4	M, R, N, S, C, U1, U2	U4	7	C-G→G-C

a Two lengths (i.e., 11 and 9 nt) were observed in the neck region of the stem-loop. Based on the nature of the nucleotides, segments were also categorized in each nanovirus along with the site number and nucleotide changes.

### Molecular modeling of stem-loop segments.

**FIG 3:**
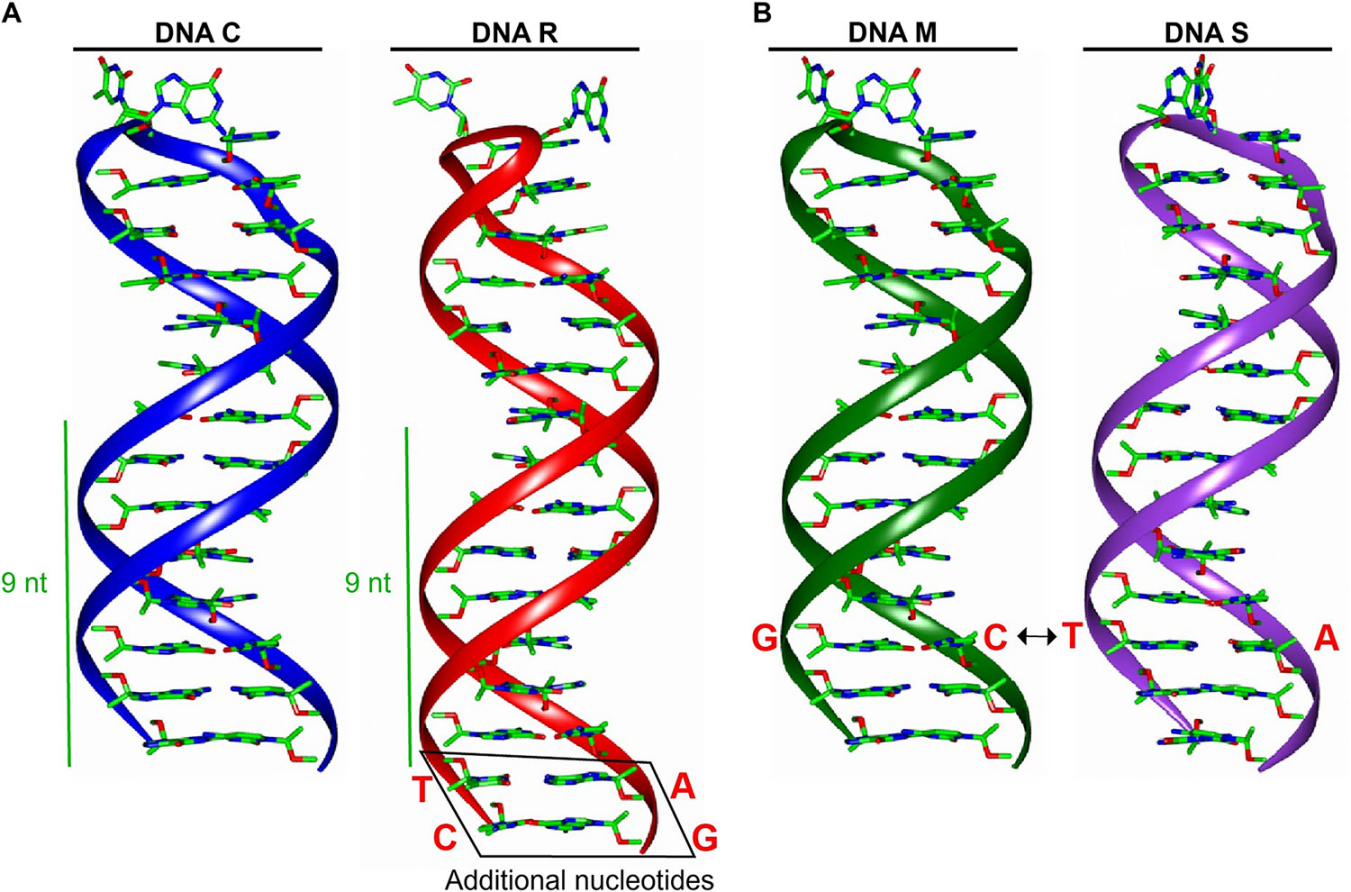
Molecular modeling of stem-loop segments. Shown is the architectural layout of the stem-loop region (CR-SL) of all segments by applying the schematic approach of PyMOL, which designed the overall shape of each subunit. (A) Structure of DNA C, U1, and U2 segments with 9-nt base pairing in the neck region of the stem-loop; (B) structure of DNA R, S, M, N, and U4 segments with 11-nt base pairing; (C) structure of DNA C, R, S, M, U1, U2, and U4 segments with G-C pairing at position 7 in the neck region of the stem-loop; (D) structure of DNA N and S segments with T-A pairing instead of G-C pairing at position 7 in the neck region of the stem-loop.

Stem-loop structure (CR-SL) containing three short repeated sequences were modeled by adding and switching of nucleotides in the neck region, which were named DNAs C, R, M, and S, respectively (Fig. 3A and B). We examined the secondary structure and found that sequence of the predicted stem-loop structure variations in the length of the stem-loop neck region and nucleotide pairing of DNAs M and S at position 7, respectively, make them different from each other. Modeled stem-loop structures were refined by removing steric clashes and bad contacts between the atoms.

### MD simulation.

**FIG 4:**
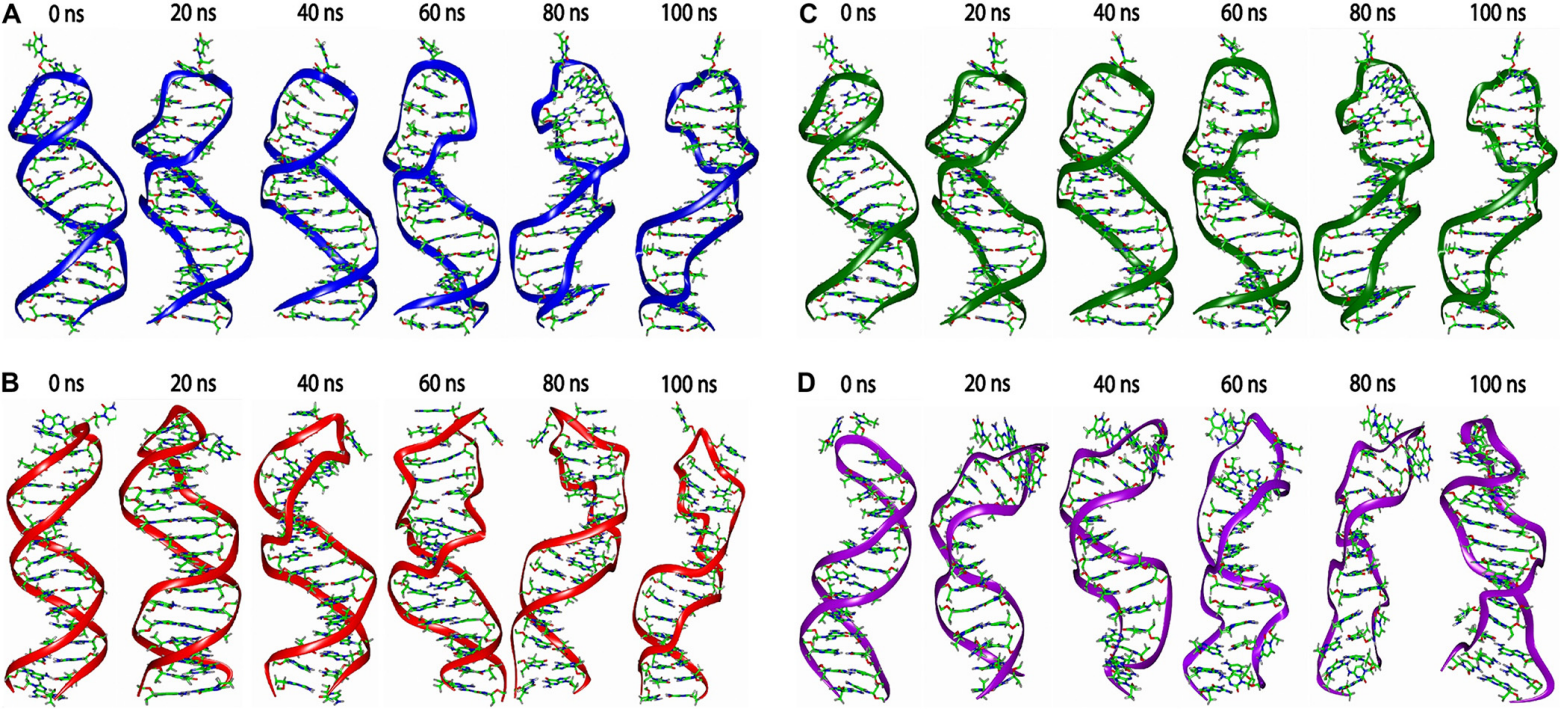
Dynamics of stem-loop structures of DNAs C, R, and S calculated by AMBER18. Simulation trajectories revealed the structural stability of the stem-loop structure at 100 ns by calculating the dynamic behavior of original and mutant stem-loop models. All calculations were observed under explicit solvent at 300 K. (A and B) Structure of mutant DNAs C and R with lengths of the neck regions of 11 and 9 nt, respectively. (C and D) Structure of mutant R and S with T-A and G-C pairing, respectively, at position 7 in the neck region of the stem-loop.

**FIG 5:**
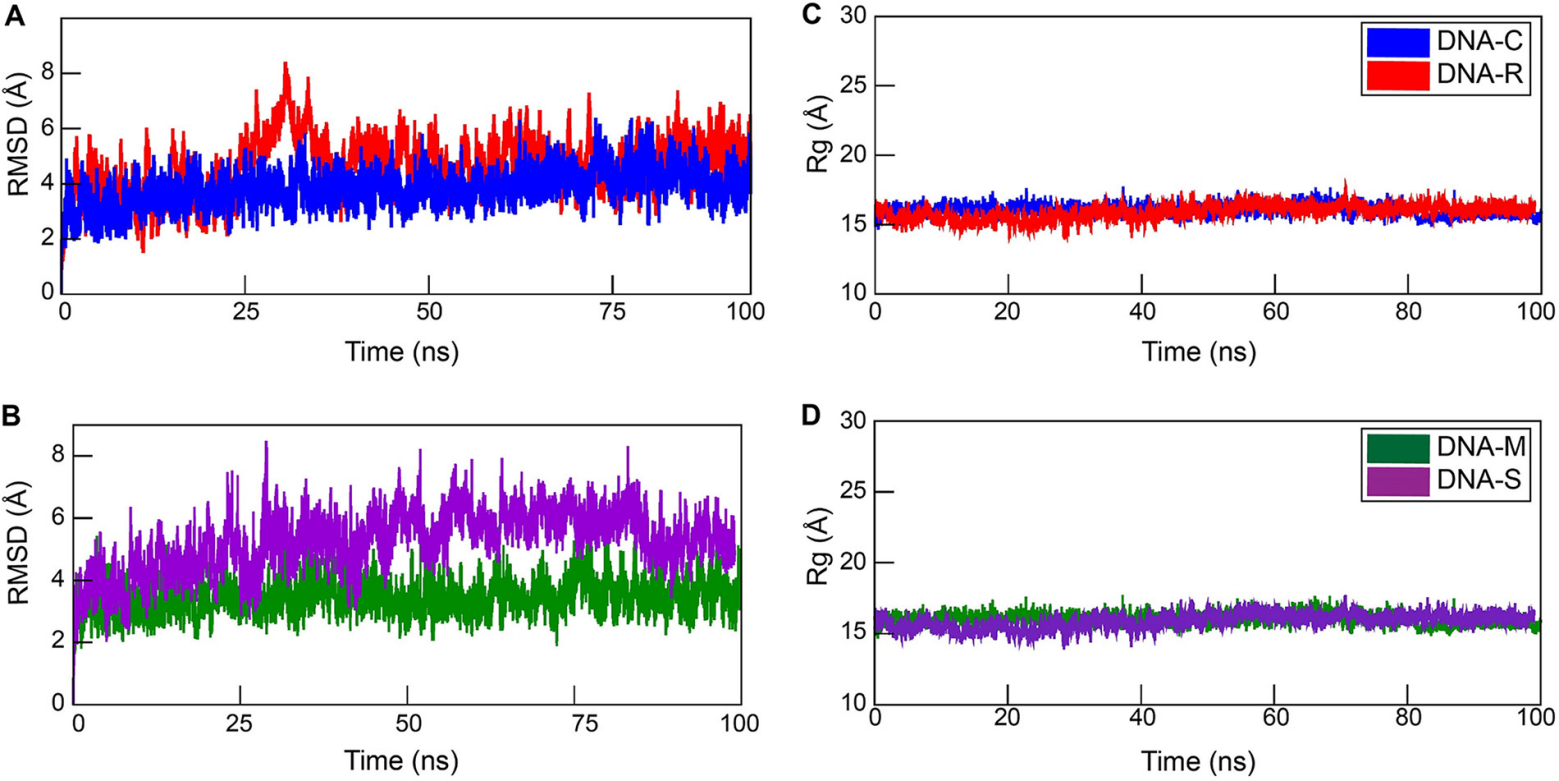
Structural and conformational analysis of the original segments versus mutants. The average RMSD and Rg of (A and C) DNA C and DNA R with length variations during 100 ns were measured as well as for mutants (B and D) DNA M and DNA S. This indicates that the overall original systems of virus DNA R and S showed stable behavior compared to mutant systems throughout the simulation time.

**FIG 6:**
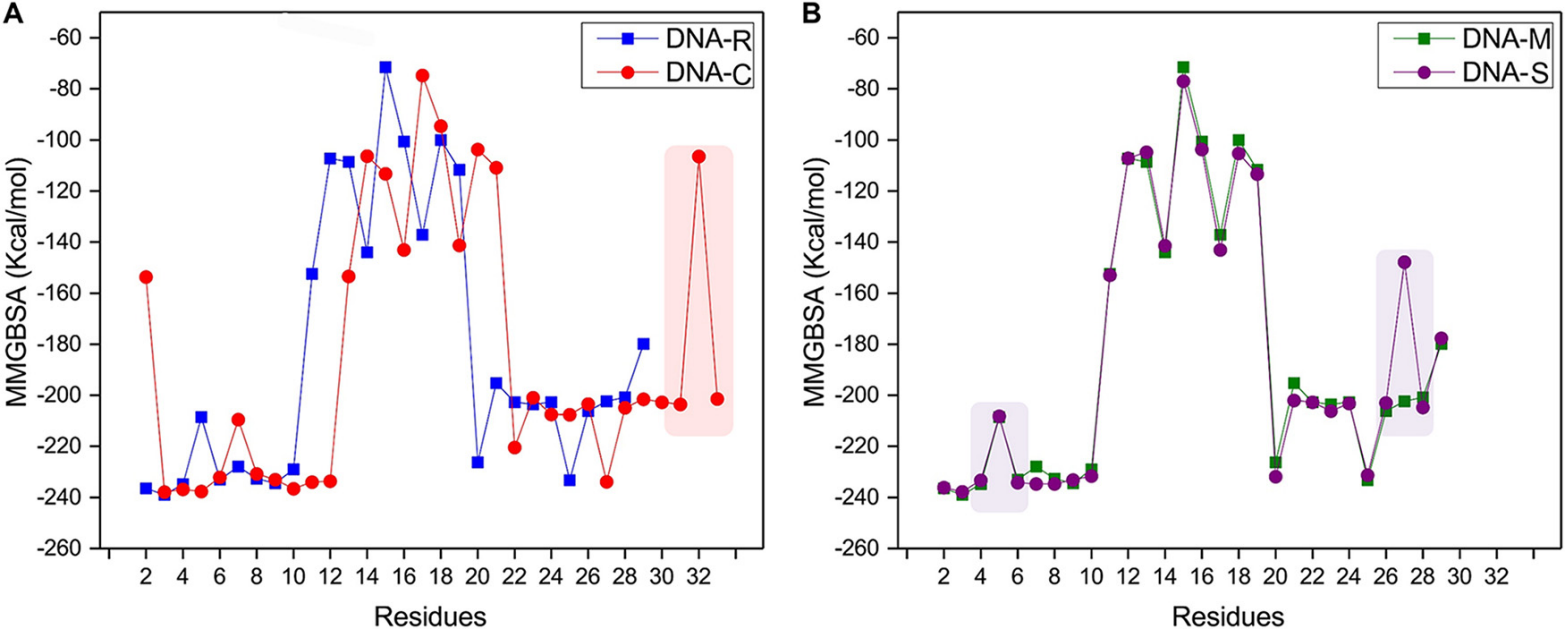
Residue energy calculations of mutants of MDV segments using MMGBSA analysis. Structural stability of the stem-loop structures by calculating the binding free energy of each residue with a focus on (A) DNA C and R with different lengths of the neck region and (B) DNA R and S with base pairing variations at position 7 of the neck region of the stem-loop.

Simulation trajectories revealed the structural stability of the stem-loop structure at 100 ns by calculating the dynamic behavior of DNAs C, R, M, and S stem-loop models (Fig. 4). All calculations were observed under explicit solvent at 300 K. The calculated root mean square deviation (RMSD) from first to last simulated trajectories provided the structural and conformational information of the systems. The average RMSDs of DNA C (9-nt pairings) and DNA R (11-nt pairing) were observed at 3.85 and 4.64 Å, respectively (Fig. 5A). We anticipated that higher RMSDs in DNA R are mostly because of additional nucleotides in the neck region that showed deviation at the 100-ns simulation in comparison to DNA C. It is also observed that RMSD of DNA M showed lower deviation than DNA S, with RMSD values of 3.45 and 5.26 Å, respectively (Fig. 5B). Overall shape and compactness of the stem-loop segments were determined by the radius of gyration (Rg) (Fig. 5C and D). The maximum radii of gyration for DNA C of 17 Å and DNA R of 19 Å were observed during 100 ns, while radii for mutant DNA M of 17 Å and DNA S of 18 Å were measured. This indicates that the overall original systems of virus DNAs C and M showed stable behavior compared to those of DNAs R and S, respectively, throughout the simulation time. We also examined the contribution of the structural stability of the stem-loop structures by calculating the binding free energy of each residue (Fig. 6). The per-residue (decomposition) analysis revealed that the loop regions of all the structures showed much higher energy than the neck region. The base pairing interactions between bases in the systems’ neck region are responsible for the smaller energy change, which maintains the neighboring interactions among the bases and stabilizes the systems.

### Infectivity of mutant ICs through agroinoculation.

**FIG 7:**
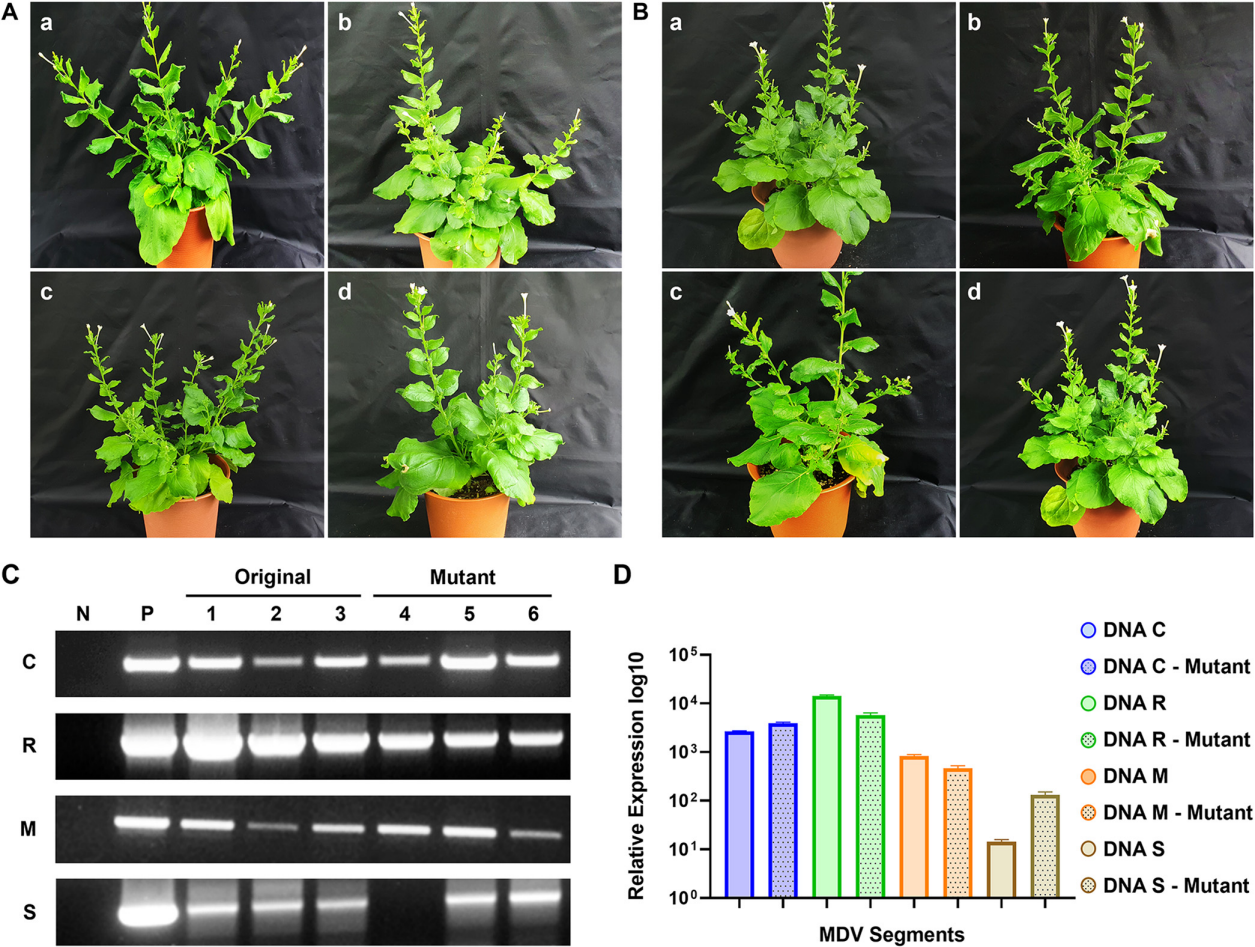
Development of symptoms in inoculated plants and their detection through PCR, followed by expression analysis through qPCR. (A) Development of symptoms in N. benthamiana by DNA C and R (with different lengths of the neck region) 28 dpi. Specifically, the symptoms that developed were evaluated and compared for the four variations: (a) original DNA C, (b) mutant DNA C, (c) original DNA R, and (d) mutant DNA R. (B) Development of symptoms in N. benthamiana by DNA R and S with base pairing variations at position 7 of the neck region of the stem-loop (a) original DNA R, (b) mutant DNA R, (c) original DNA S, and (d) mutant DNA S 28 dpi. (C) Confirmation of the segments in N. benthamiana through PCR; (D) analysis of expression of the segments in the infected N. benthamiana.

N. benthamiana plants showed dwarfism and bushy symptoms in both original and mutant IC cases but with different severity (Fig. 7A and B). PCR was processed to investigate viral infections in infected N. benthamiana plant samples. The virus segments both original and mutants of DNAs C, R, M, and S were detected in all samples, except the one inoculated with the DNA R mutant, among three samples (Fig. 7C). The virus reconstituted in N. benthamiana maintained the exact nucleotide sequence of the original clone.

### Expression analysis through qPCR.

The relative expression levels of the mutant and original segments were analyzed. The expression of the DNA C mutant (11-nt pairings) was higher than that of the original DNA C (9-nt pairings), whereas the DNA R mutant (9 nt pairings) was expressed relatively lower than the original DNA R (11 nt pairings). Similarly, the DNA M mutant based on site-specific mutation by switching nucleotides at a specific point from G-C to T-A showed a bit lower expression than the original DNA M. DNA S mutant segment with configuration T-A and G-C expressed a bit higher than the DNA S with configuration T-A (Fig. 7D).

## DISCUSSION

Due to their multicellular way of life, nanoviruses are confusing but intriguing (26). Nanoviruses have multiple segments localized in various compartments that are not mandatory to be together to cause infectivity (27). These segments with specific functions can carry out the function for the other segment (e.g., DNA R can cause replication of all other segments, and the intergenic region [IR] plays an essential role in this regard). The IR comprises repeated sequences of iterons that provide the site to bind replication protein and other proteins, respectively (28, 29). The CR-SL in the IR with nonanucleotide TAGTATTAC provides a nicking site for replication. Like geminiviruses, several *cis*-elements and transcription factor binding sites might be located in the nanovirus IR. The G box and TATA box mutant studies in tomato golden mosaic virus demonstrated that these motifs are essential for transcription and affect the origin of replication activity (30). Previous studies characterized MDV in South Korea, Vietnam, and Taiwan (31, 32), with a difference in SL-CR among the segments. All segments of MDV when aligned revealed the differences in the segments. DNAs C, U1, and U2 were slightly different from DNAs S, R, M, N, and U4, as the number of nucleotides capable of pairing in the neck region was less in DNAs C, U1, and U2 than those in other segments. DNAs C, U1, and U2 possess 9-nt pairings, whereas DNAs S, R, M, N, and U4 have 11-nt pairings. Another difference in the nucleotide pairing at position 7 in the neck region of stem-loop was noticed, where DNAs N and S have a T-A pairing, whereas DNAs C, S, R, M, U1, U2, and U4 contain a G-C pairing. The expression levels of DNAs C, U1, and U2 are more identical than those of other segments, whereas DNAs N and S were expressed at very low (almost identical) levels in infected papaya samples. These identical expression levels made us think there might be a possible linkage between expression level and the composition/nature of the IR, especially the stem-loop region. Both of these variations (i.e., length and composition/nature of pairing) were noticed in other nanoviruses as well (although not exactly the same), including FBNSV, FBNYV, BMLRV, PNYDV, etc., as shown in Fig. S1A to G. These variations also lead to the difference in motif formation and, as a result, possible differences in protein binding sites. The impact of these variations was attempted to investigate using bioinformatics and wet-lab approaches in this study. First, by *in silico* studies, we examined the secondary and tertiary structures and found that sequence of the predicted stem-loop structure variations in the length of the stem-loop neck region of DNAs C and R and nucleotide pairing of DNAs M and S at position 7, respectively. The MD simulations have been used to determine the stability of macromolecules by predicting the time-dependent behavior (33). The structural and conformational stability of the stem-loop structure is important to determine the functional changes in the host plant. In MD simulations, the mutated segments of stem-loop structure were expected to alter the conformational flexibility that can change the expression of the virus segments. The mutated segments of DNAs C and R (based on length of the neck region of the stem-loop) and DNAs M and S (based on nature of base pairing at position 7) were constructed by either by adding or deleting the respective nucleotide pairing in both segments, as mentioned in Materials and Methods. Infectious clones in both sets (original and mutant) were inoculated using the pinprick method in N. benthamiana to check their impact. We found that the addition of nucleotide sequences in the neck region increases the expression of the virus, whereas the removal of nucleotides has the opposite effect. The DNA C mutant with an 11-nt base pairing length shows higher expression than DNA C with a 9-nt base pairing length. DNA R when mutated to a 9-nt base pairing length has an expression level lower than the original DNA R. The DNA M mutant with an A-T base pairing at position 7 had lower expression DNA M with the G-C base pairing. The expression level of the DNA S mutant with G-C base pairing was higher than that of DNA S with the A-T base pairing. These variations show the stem-loop composition as an important factor in controlling the expression level of the virus segments, but this still needs further investigation.

## MATERIALS AND METHODS

### Nucleotide sequences and their analysis.

The sequences of all segments of the reported members of genus *Nanovirus* were retrieved from GenBank (http://www.ncbi.nlm.nih.gov). First, all sequences were aligned in the same position, mainly with the nonanucleotide (5′-TAATATT/AC-3′) at the nicking position. Then, using the Qiagen CLC sequencer view 8.0 (https://digitalinsights.qiagen.com/), open reading frames (ORFs) and intergenic regions (IRs) were separated and aligned to the ORFs and IRs of other segments of the same and other nanoviruses, respectively.

### Characterization of segments based on stem-loop structure composition.

All genomic DNAs contain conserved inverted repeat sequences, potentially forming a stem-loop structure (CR-SL) containing three short repeated sequences (iterons). Therefore, IRs, especially CR-SL of all segments of nanoviruses, were analyzed carefully, and segments with different stem-loop structure compositions were identified within nanoviruses. Furthermore, motifs were analyzed in the IRs of MDV and FBNYV by using PLACE software (34) and The Arabidopsis Information Resource (TAIR).

### Molecular modeling of the stem-loop segment.

To model the architectural layout of the stem-loop segments (CR-SL), we applied the schematic approach of PyMOL (35), which designed the overall shape of each subunit. To investigate the effect of nucleotide variations on the stability and flexibility of the stem-loop structure, we added and switched nucleotides in DNAs R and S, respectively, using WinCoot (36). Refinement of all the stem-loop model structures was done in UCSF Chimera (37).

### MD simulations.

The dynamics of stem-loop structures were calculated by AMBER18 (38). Topologies of the system were built using the AMBER force field (39). All systems were placed in a simulative box, with periodic boundary conditions, filled with TIP3P water molecules. All simulative systems were kept at least an 8-Å distance from the box border. The bad contacts and steric clashes of each system were minimized in two steps. In the first step, restraint was applied to the stem-loop structure, and minimization was performed on the water and ions; in the second step, the entire stem-loop structure was minimized with 2,500 steps without any restraints. After minimization, all systems were gradually heated up from 0 to 300 K by applying nuclear magnetic resonance restraints over a time scale of 20 ps. In the next stage, equilibrations were carried out with a time step of 100 ps at a constant temperature of 300 K and constant volume.

Equilibrations continued at a constant temperature of 300 K and a constant pressure of 1 atm, whereas constraints were removed. MD simulations were performed for 100 ns, and the coordinates were saved every 0.5 ps. The structural and conformational analysis of all systems was conducted by VMD (40). MD simulation trajectories were analyzed by the CPPTRAJ module (41) of AMBER to analyze the root mean square deviation (RMSD) and radius of gyration (Rg).

### Energy calculation.

The energy contribution of per-residue decomposition was conducted by the molecular mechanics/generalized Born surface area method using AMBER18 (42). MD simulation trajectories were utilized to calculate per-residue decomposition, which calculates the energy contribution of single residues by summing its interactions over all residues in the system. Graphical representations were made using Grace software.

### Mutant construction based on the stem-loop length and composition.

Based on the stem-loop composition, mutants of MDV segments were designed and constructed by using the Q5 site-directed mutagenesis kit (NEB, MA, USA). Variations observed in the stem-loop region were categorized in two aspects: (i) the length of the neck region and (ii) the composition of the neck region. In the former, two genomic DNAs (i.e., DNA C, with 9-nt pairings, and DNA R, with 11-nt pairings) were mutated by adding and removing 4 nt in a way that they either made or removed two extra pairings, respectively (Fig. S2A to D). In the latter, DNAs M and S were mutated based on site-specific mutation by switching nucleotides at a specific point from T-A to G-C and G-C to T-A, respectively.

### Infectious clone construction and agroinfiltration.

Infectious clones (ICs) (1.1mer) of the mutated DNA segments (DNA C, R, M and C, as mentioned above) were constructed to check their infectivity and impact variance in the host plants. All original MDV segments were already constructed and available in the authors’ laboratory. To make an IC of mutant virus, the same method of IC construction with slight modifications was used as in the case of geminiviruses (43, 44). Two partial genomes of each virus sequence containing restriction sites at the edges were amplified to make infectious clones using primer sets based on the extracted sequence as shown in schematic diagram of MDV DNA R (Fig. S3A and B). According to the manufacturer’s instructions, these partial genomes were ligated into the pGEM-T Easy vector (Promega, USA) using the T/A cloning technique, followed by sequencing (Macrogen, South Korea) and restriction digestion using specific enzymes. The two partial genomes were introduced into the pCAMBIA1303 vector and first transformed into competent Escherichia coli strain DH5α using the heat shock method and then into the GV3101 *Agrobacterium* strains. GV3101 *Agrobacterium* strains (transformed and untransformed) were cultured in Luria-Bertani broth in the presence of a pCAMBIA1303 selection antibiotic, such as kanamycin (50 mg/L), and strain-specific selection antibiotics, such as gentamicin and rifampin (50 mg/L), at 28°C with agitation for 30 h (until the optical density at 600 nm [OD_600_] was 0.8 to 1.0). Before inoculation, all segments were collected in a 50-mL tube in the same amount (i.e., 2 mL), followed by centrifugation and resuspension in an infiltration buffer (10 mM MES [morpholineethanesulfonic acid] [pH 5.6], 10 mM MgCl_2_, and 100 μM acetosyringone) to a final OD_600_ of 0.6 to 0.8, and incubated for 3 h at room temperature in the dark. This method was applied to the original and mutated MDV genomes. Agroinoculation was performed by pinpricking (45) in ~4- and 6-week-old N. benthamiana plants. Each virus was inoculated in 3 N. benthamiana plants along with mock plant inoculation with the GV3101 *Agrobacterium* strain only.

### PCR and qPCR analysis.

Leaf tissue samples were collected from mock-infected and infected plants 28 days postinoculation (dpi) to check infectivity through PCR processing using the primers DNA C/F (5′-CTCCCATCTTCCTGAAGAATTG-3′), DNA C/R (5′-AACAACTCTCCACATGAGAGGT-3′), DNA R/F (5′-CGATTAGTTCCAGGAGCCCA-3′), DNA R/R (5′-ATGGCTTCCTCTGCAACTCC-3′), DNA M/F (5′-GGACCCTGGATATTATCAAGGT-3′), DNA M/R (5′-CCACCATTGGATGGCTGACT-3′), DNA S/F (5′-AATGGGATGAAGAGACGACGAA-3′), and DNA S/R (5′-CATTGGACTCAACCTTGCCTTT-3′), which were specifically designed to amplify these segments each with a target size of ~300 to 350 bp.

The expression of each segment (original and mutant) was tested by qPCR as well. Reactions were performed using the SYBR premix *Ex Taq* (*Tli* RNase H Plus; TaKaRa, Shiga, Japan) with specific primer sets (see Table S1 in the supplemental material). Cycling of PCR consisted of predenaturation at 95°C for 5 min, followed by 40 cycles of a denaturation step at 95°C for 10 min, an annealing step at 60°C for 15 s, and an extension step at 72°C for 20 s using a Rotor Gene Q thermocycler (Qiagen, Hilden, Germany). The annealing temperature was modified following the melting temperature of each primer, and each reaction was repeated at least three times. Data analyses were conducted by the threshold cycle (2^−ΔΔ^*^CT^*) method (46).
