# Supplementary material for: Insights into the Differential Composition of Stem-Loop Structures of Nanoviruses and Their Impacts
Source: Microbiol Spectr. 2023 Jun 27;11(4):e04798-22. doi: 10.1128/spectrum.04798-22 (PMC10434203; doi:10.1128/spectrum.04798-22)
Supplement: Supplemental file 1 — Supplemental material. Download spectrum.04798-22-s0001.pdf, PDF file, 0.7 MB [file spectrum.04798-22-s0001.pdf]

Supplementary data

S1

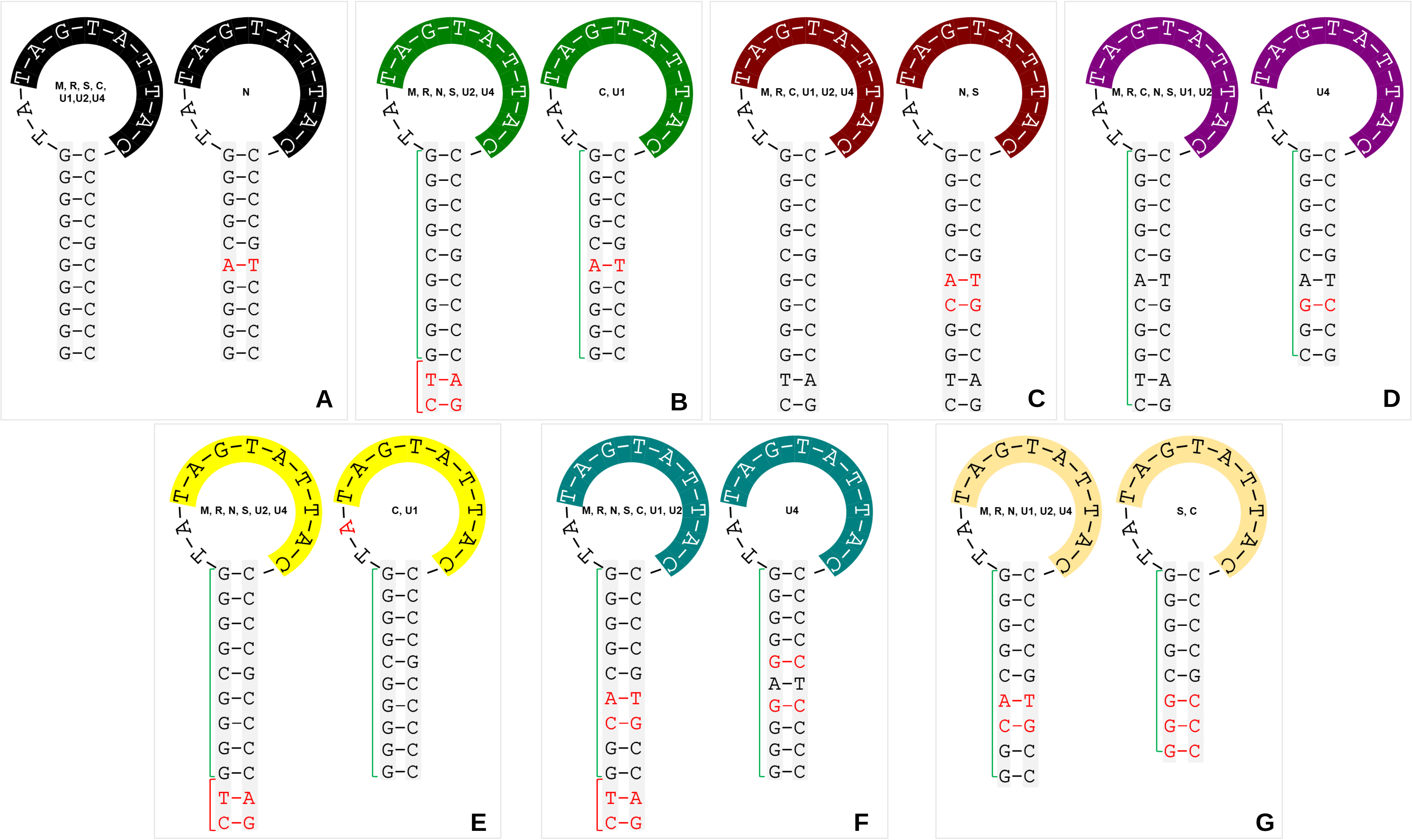
M, R, S, C, U1,U2,U4
N
A
M, R, N, S, U2, U4
C, U1
B
M, R, C, U1, U2, U4
N, S
C
M, R, C, N, S, U1, U2
U4
D
M, R, N, S, U2, U4
C, U1
E
M, R, N, S, C, U1, U2
U4
F
M, R, N, U1, U2, U4
S, C
G

Fig. S1. Variations in the stem-loop region of DNA segments of nanoviruses. Nanoviruses: faba bean necrotic stunt virus, faba bean necrotic yellows virus, black medic leaf roll virus, pea necrotic yellow dwarf virus, faba bean yellow virus, cow vetch latent virus, and subterranean clover stunt virus (7/9 nanoviruses) have shown the variations in the stem loop region.

S2

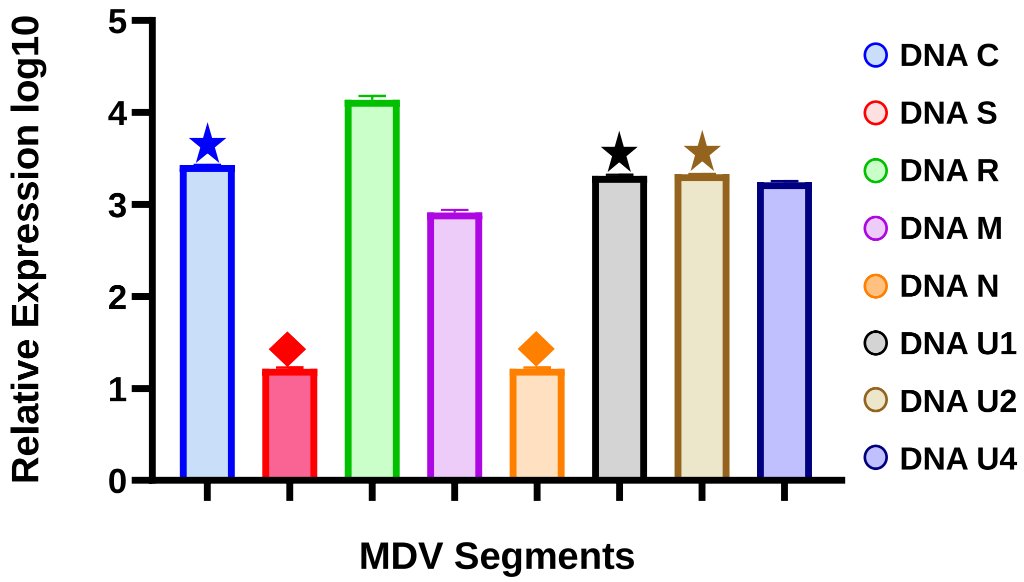
Relative Expression log10
5
4
3
2
1
0
MDV Segments
DNA C
DNA S
DNA R
DNA M
DNA N
DNA U1
DNA U2
DNA U4

Fig. S2. qPCR analysis of viral segment expression of MDV infection in papaya. DNA C, U1, and U2 show almost the same expression level as the other segments. These three segments have 9 base pairings in the neck region (Aspect 1) and are highlighted in the red color boxes. Segments N and S show the same expression level (i.e., lower than the others), as both these segments have different base pairings [i.e., T–A instead of G–C (Aspect 2)] and are highlighted in the blue boxes.

S3

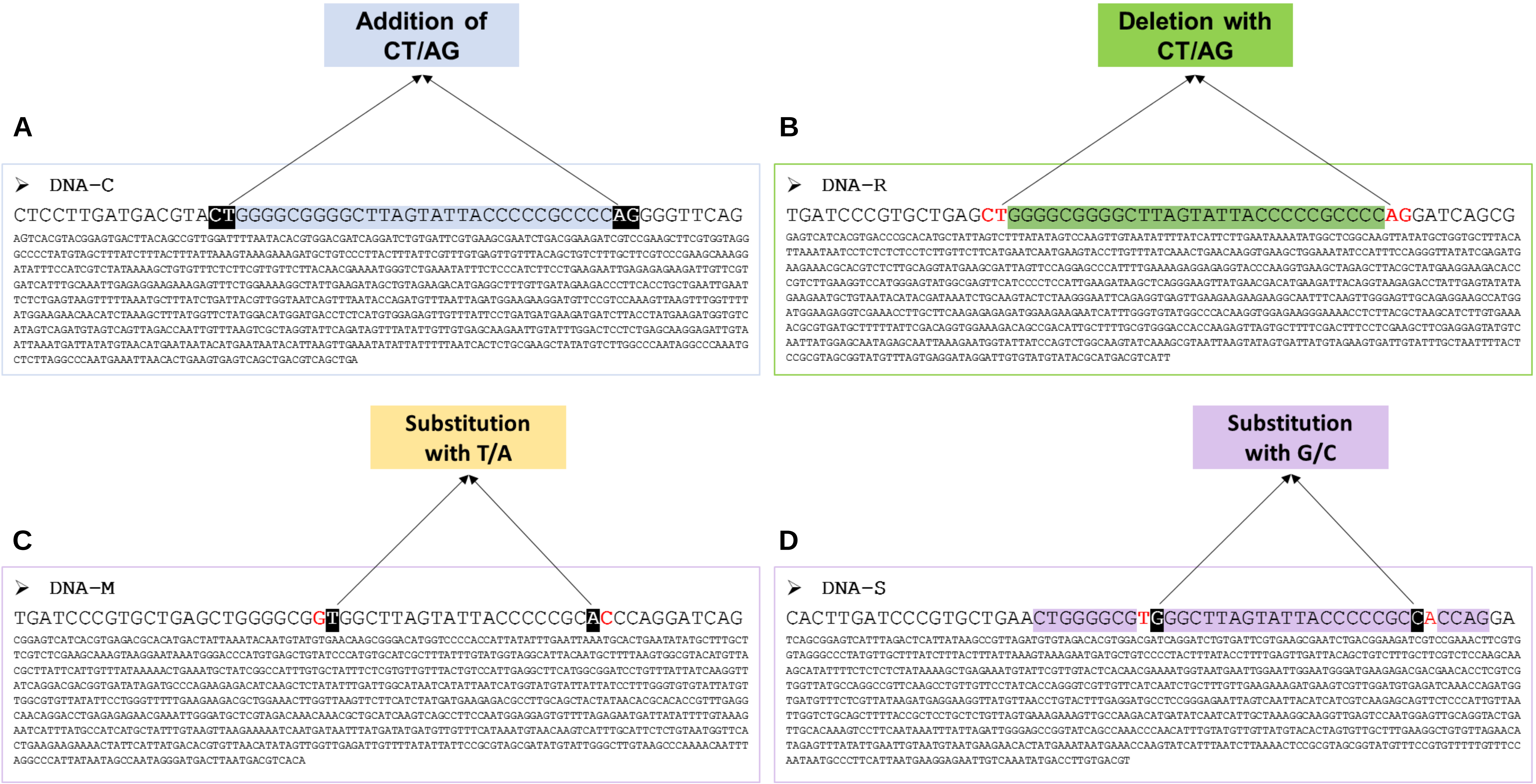

Addition of CT/AG
Deletion with CT/AG
A
B
DNA–C
CTCCTTGATGACGTACTGGGGCGGGGCTTAGTATTACCCCCGCCCCAGGGGTTCAG
DNA–R
TGATCCCGTGCTGAGCTGGGGCGGGGCTTAGTATTACCCCCGCCCCAGGATCAGCG
Substitution with T/A
Substitution with G/C
C
D
DNA–M
TGATCCCGTGCTGAGCTGGGGCGGTGGCTTAGTATTACCCCCGCACCCAGGATCAG
DNA–S
CACTTGATCCCGTGCTGAACTGGGGCGTGGCTTAGTATTACCCCCGCCACCAGGA

Fig. S3. qPCR analysis of viral segment expression of MDV infection in papaya. DNA C, U1, and U2 show almost the same expression level as the other segments. These three segments have 9 base pairings in the neck region (Aspect 1) and are highlighted in the red color boxes. Segments N and S show the same expression level (i.e., lower than the others), as both these segments have different base pairings [i.e., T–A instead of G–C (Aspect 2)] and are highlighted in the blue boxes.

S4

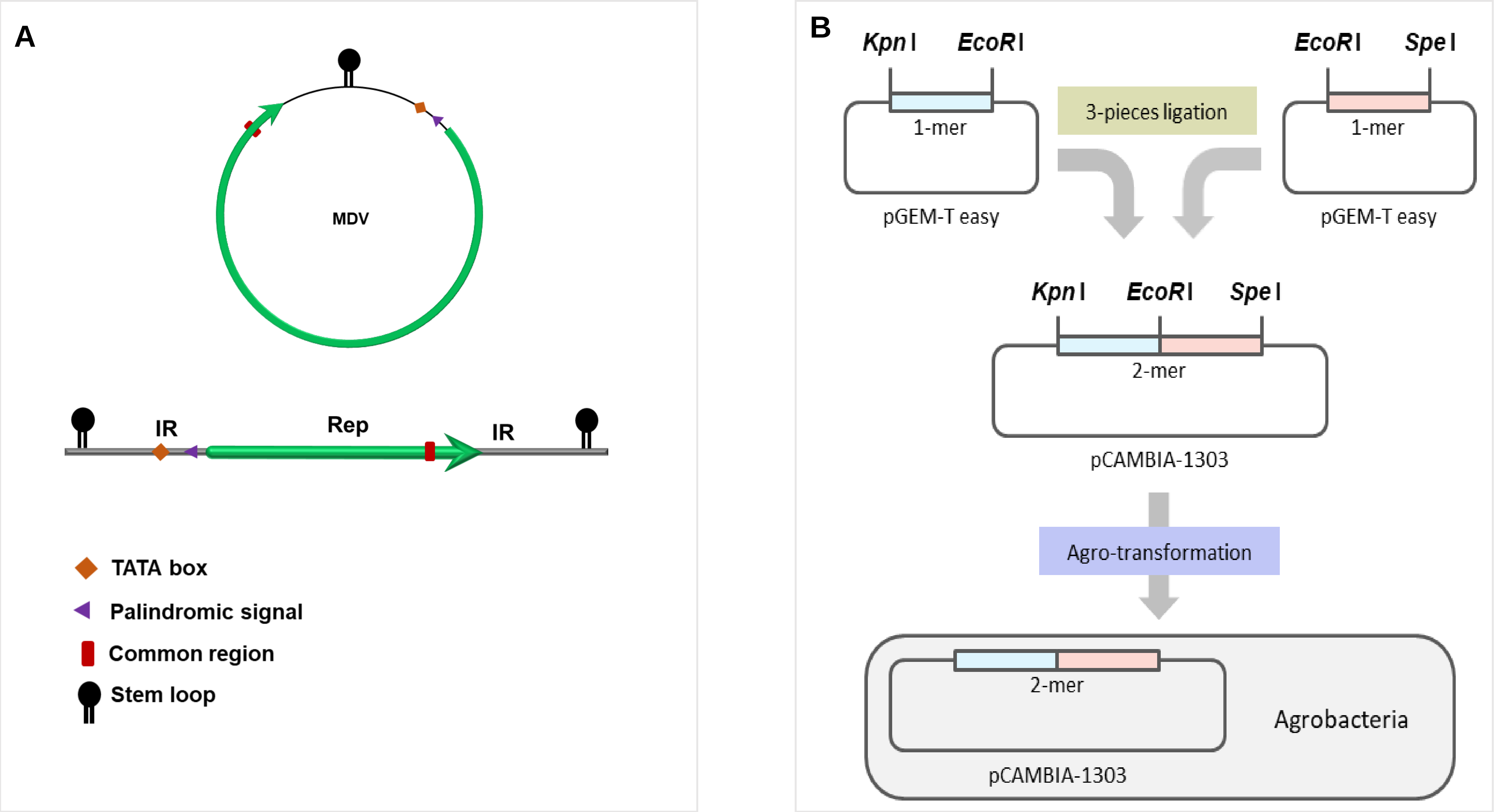
A
MDV
IR
Rep
IR
TATA box
Palindromic signal
Common region
Stem loop
B
Kpn I
EcoR I
1-mer
pGEM-T easy
3-pieces ligation
EcoR I
Spe I
1-mer
pGEM-T easy
Kpn I
EcoR I
Spe I
2-mer
pCAMBIA-1303
Agro-transformation
2-mer
pCAMBIA-1303
Agrobacteria

Fig. S4. Schematic diagram of IC construction of Nanoviruses. (A) An additional IR region has been added along with the full sequence of MDV segment. (B) The method to construct 1.1 mer IC of DNA R has been shown. Restriction enzyme sites i.e., KpnI at the start of IC1 and SpeI at the end of IC2 are added . EcoRI is the common point of digestion (end of IC1; start of IC2) existing naturally in the sequence. The sequences of both IC1 and IC2 have been shown as well in the box on the right side. Restriction enzymes in the sequences (IC1 and IC2) are shown in bold letters. Both IC1 and IC2 are ligated with digested pCambia-1303 followed by the transformation into Agrobacterium strain GV3101.

Supplemental Table S1. Primer sets used for expression analysis through qPCR in this study.

Primer name	Sequence (5′ – 3′)	Target size
MDV R-F	GGCTTAGTATTACCCCCGCC	137
MDV R-R	GCACCAGCATATAACTTGCCG	
MDV M-F	GGATCCGCGGAGACATCAC	179
MDV M-R	GAAGGCACATGGGTTACAGC	
MDV C-F	AATACGCGTGGACGATCAGG	177
MDV C-R	CGGGAAGAAGCAAAGACAGC	
MDV N-F	GGAAGTTTGTTGTGTCCGTTCT	180
MDV N-R	CATACCGCTACGCGGAGTAA	
MDV S-F	CCGGTATCAGCCAAACCCAA	164
MDV S-R	ATACCGCTACGCGGAGTTTT	
MDV U1-F	CTTCGTCTCGAAGCAAAGGAC	145
MDV U1-R	TCGTTCGCAGACATAACCTCAA	
MDV U2-F	AAGGAAGAACAAGATGCTTTCTGG	150
MDV U2-R	TCTAAGAACCCACCGTGCAG	
MDV U4-F	TGCAACACTATTGTTTGTGTGGT	172
MDV U4-R	GAACCTGGGTTCCATAGGCA	
